# Active Vitamin D Ameliorates Arsenite-Induced Thyroid Dysfunction in Sprague–Dawley Rats by Inhibiting the Toll-like Receptor 4/NF-KappaB-Mediated Inflammatory Response

**DOI:** 10.3390/toxics12120887

**Published:** 2024-12-06

**Authors:** Hui Li, Jie Xiang, Qian Song, Ying Jin, Meitong Zhou, Lili Fan, Dapeng Wang

**Affiliations:** 1 Key Laboratory of Environmental Pollution Monitoring and Disease Control, Ministry of Education, School of Public Health, Guizhou Medical University, Guiyang 561113, China; huihuier129@163.com (H.L.); 18892341510@163.com (J.X.); sqhalo@163.com (Q.S.); jinying199926@163.com (Y.J.); zhoumeitong0223@163.com (M.Z.); 2 Collaborative Innovation Center for Prevention and Control of Endemic and Ethnic Regional Diseases Co-Constructed by the Province and Ministry, Guizhou Medical University, Guiyang 561113, China

**Keywords:** sodium arsenite, thyrotoxicity, vitamin D, thyroid hormone, TLR4/NF-κB signaling pathway

## Abstract

Arsenic, a well-known environmental endocrine disruptor, exerts interference on the body’s endocrine system. Our previous investigations have demonstrated that chronic exposure to sodium arsenite (NaAsO_2_) can induce thyroid damage and dysfunction in Sprague–Dawley (SD) rats. Vitamin D (VD) is an indispensable fat-soluble vitamin that plays a crucial role in maintaining thyroid health. In recent years, numerous studies have demonstrated the association between VD deficiency and the development of various thyroid disorders. However, the precise intervention roles and mechanisms of VD in arsenic-induced thyroid injury remain elusive. This study aimed to investigate the intervention effect of VD on NaAsO_2_-induced thyroid dysfunction in SD rats. The results demonstrated that exposure to NaAsO_2_ activates the TLR4/NF-κB signaling pathway in thyroid tissue of rats, leading to apoptosis of thyroid cells and subsequent inflammatory damage and disruption of serum thyroid hormone secretion. Supplementation with TAK-242 (a TLR4 inhibitor) and VD effectively inhibits the activation of the TLR4/NF-κB signaling pathway in rat thyroid tissue exposed to NaAsO_2_, thereby reducing the inflammatory damage and dysfunction caused by arsenic exposure. In conclusion, the findings of this study offer innovative insights into the application of VD in the prevention and treatment of thyroid dysfunction caused by arsenic exposure.

## 1. Introduction

The metalloid element arsenic can be detected in both organic and inorganic forms within the environment [1]. The exposure of humans to arsenicals occurs through multiple pathways, encompassing ingestion via food, consumption of drinking water, and inhalation of airborne particles [2,3]. Recent studies have unveiled that exposure to inorganic arsenic not only elicits multi-organ damage, including the skin, liver, kidneys, and lungs [4], but also disrupts the endocrine system by exerting influence on various hormones and their receptors. The disruption of endocrine homeostasis can trigger the initiation and advancement of various diseases [5]. The thyroid has the largest endocrine gland in the body, and its function is regulated by the hypothalamic–pituitary–thyroid (HPT) axis. The hypothalamus releases the thyrotropin-releasing hormone (TRH), which is transported to the adenohypophysis through the hypothalamic–pituitary portal system to promote the synthesis and release of thyroid hormones (THs) [6]. It coordinates the synthesis, secretion, transportation, and metabolism of triiodothyronine (T_3_) and tetraiodothyronine (T_4_) to maintain the dynamic balance of THs [7]. Under normal circumstances, the thyroid gland uptakes inorganic iodine from the bloodstream through the sodium-iodide symporter (NIS) when stimulated by the thyroid-stimulating hormone (TSH) secreted by the pituitary gland. Thyroid peroxidase (TPO) simultaneously activates iodine and catalyzes the coupling of thyroglobulin (TG) to generate biologically active free T_3_ (FT_3_) and free T_4_ (FT_4_) [8]. With the advancement of research, the correlation between environmental arsenic exposure and thyroid damage has attracted growing attention. Epidemiological studies conducted in various countries consistently demonstrate significant associations between levels of environmental arsenic exposure and serum concentrations of THs, as well as the secretion of TSH [9,10]. A birth cohort study from China has also revealed that maternal exposure to a low-level of arsenic during pregnancy directly affects neonatal thyroid hormone secretion, independent of maternal arsenic exposure effects [11]. In vivo experiments have demonstrated that arsenic could accumulate in animal thyroid tissue, disrupting THs secretion and causing thyroid tissue damage, thus impairing its function and exerting thyrotoxic effects [12,13]. Nandheeswari [14] exposed female mice to NaAsO_2_ for 30 days, revealing that arsenic can disrupt the permeability of thyroid lysosomes, leading to increased TSH levels and decreased THs levels. These effects also impact follicular development patterns in female mice. Our latest reports indicate that prolonged exposure to NaAsO_2_ (36 weeks) resulted in significant damage to the thyroid tissue of SD rats, with extensive apoptosis observed in thyroid cells. This was conducted by a marked decrease in serum THs levels and a significant increase in TSH levels [15,16]. However, the precise molecular mechanism underlying thyroid damage caused by arsenic exposure remains elusive and the targeted therapeutic strategies for arsenic-induced thyrotoxicity have yet to be explored in the current literature.

Toll-like receptor 4 (TLR4) is a class of pattern recognition receptors expressed in various immune cells and performs a crucial function in the innate immune system [17]. Upon stimulation by ligands, TLR4 binds to myeloid differentiation factor 88 (MyD88) in order to trigger a signal cascade, resulting in the activation of nuclear factor-κB (NF-κB) and the production of pro-inflammatory cytokines, which are crucial for immune responses and inflammatory processes [18]. Currently, research has shown that the TLR4/NF-κB signaling axis may exert a significant influence on the development of thyroid disorders caused by various factors. Peng et al. [19] discovered a significant up-regulation of TLR4 signal transduction levels in peripheral blood monocytes among individuals diagnosed with autoimmune thyroid diseases. Subsequently, Liu et al. [20] observed significant elevated expression levels of TLR4, and NF-κB subunits (including p65, p50, p-P65, and p-P50) in human thyroid papillary tissue compared to benign thyroid hyperplasia tissue. In addition, the expression levels of TG, TPO, TSH, NIS, and other thyroid function-related indicators were affected after the activation of TLR4/NF-κB signaling axis [19,21,22,23]. These studies suggest a strong link between the activated TLR4/NF-κB signaling axis and various thyroid disorders. However, it remains unclear if this signaling axis is involved in arsenic-induced thyroid injury.

Vitamin D (VD) is an essential fat-soluble nutrient that regulates calcium and phosphorus metabolism, and modulates various biological processes including immunity, cell cycle, and cellular differentiation. When exposed to ultraviolet radiation, VD converts to its active form, 1,25-dihydroxyvitamin D_3_ [1,25-(OH)_2_D_3_], which binds to the VD receptor (VDR) and exerts immunomodulatory effects while also influencing cell growth, differentiation, and apoptosis [24]. Globally, numerous countries, including India, Tunisia, Pakistan, and Afghanistan, have reported a significant prevalence of VD deficiency with more than 20% of their populations exhibiting low levels of this essential nutrient [25]. The current large-scale epidemiological studies consistently show a strong correlation between VD deficiency and various diseases, including cardiovascular disorders, endocrine conditions, and malignancies [26]. More importantly, recent population-based studies have demonstrated that VD deficiency is also significantly related to different types of thyroid disorders. For example, Mackawy et al. [27] found that hypothyroidism patients often have VD deficiency and hypocalcemia, which is significantly correlated with the severity of their hypothyroid condition. Štefanić et al. [28] discovered a higher prevalence of VD deficiency among patients with Hashimoto’s thyroiditis (HT) in comparison to healthy adults. Further research has demonstrated that VD supplementation not only improves thyroid dysfunction, but also serves as a preventative and therapeutic measure for pathological changes in thyroid tissue. It can effectively reverse the production of thyroid autoantibodies and correct cytokine imbalances [29].

Notably, recent researchers from both in vivo and in vitro studies have discovered that VD can alleviate various diseases by regulating the TLR4/NF-κB signaling axis and attenuating inflammatory response at multiple levels. Luo et al. [30] found that VD deficiency was associated with cirrhosis-induced intestinal injury. Additionally, they discovered that VD modulates the TLR4/NF-κB signaling pathway, thereby effectively mitigating intestinal inflammation and oxidative stress in cirrhotic rats. Liu et al. [31] demonstrated that high-dose VD treatment in diabetic rats could effectively reduce renal tubulointerstitial fibrosis and inflammatory cell infiltration. It also reduced the expression of TLR4, MyD88, and NF-κB proteins in rat renal cells, preventing tubulointerstitial fibrosis both in vitro and in vivo. Furthermore, VD intervention can lead to a down-regulation in the expression of TLR4, NF-κB, and TNF-α in hepatic tissue of diabetic rats, thereby mitigating liver inflammation and fibrosis [32]. However, it remains unclear whether VD intervention can ameliorate arsenic-induced thyroid damage and if its antagonistic mechanism is associated with the TLR4/NF-κB signaling pathway.

Based on the aforementioned background, this study established an animal model of NaAsO_2_-induced thyroid injury in SD rats by incorporating previous studies and utilizing the intervention model of TLR4 inhibitor TAK-242 and active VD. The objective is to systematically elucidate the specific role of the TLR4/NF-κB signaling pathway in arsenic-induced thyroid injury and investigate the antagonistic effect of VD at an in vivo animal level, thereby providing a novel strategy for effectively treating thyroid endocrine damage caused by arsenic exposure.

## 2. Materials and Methods

### 2.1. Reagents and Instruments

Information related to the reagents and instruments used in this study is briefly summarized in Supplementary Table S1.

### 2.2. Animal Grouping and Sample Collection

Twenty-four healthy weaning SD rats (three weeks of age, clean grade, weighing 80–100 g), half male and half female, were obtained from Liaoning Changsheng Biotechnology [SCXK-LN2015-0001] and housed at Guizhou Medical University (GMU) Animal Experiment Center. This study was granted approved by GMU Laboratory Animal Ethics Committee (No. 2000207). After one week of adapting to the diet (the feed ingredients and diet composition are displayed in Supplementary Tables S2 and S3), the rats were randomly divided into four groups (*n* = 6). The control group was given 10 mL/kg of normal saline orally daily, while the As^III^ group was given 10 mg/kg of NaAsO_2_ orally daily. Additionally, the TAK-242 intervention group and active VD intervention group were both administered a daily gavage of 10 mg/kg NaAsO_2_. After 12 weeks, the rats in each group, respectively, received TAK-242 (0.5 mg/kg.bw by intraperitoneal injection, three days per week) and active VD (10 μg/kg.bw by gavage, daily), simultaneously. The experimental environment should be maintained at a temperature range of 22–24 °C, with a relative humidity level between 60% and 70%, and subjected to an alternating light/dark cycle of 12 h each. After 36 weeks, all rats were anesthetized using a 0.9% solution of pentobarbital sodium. Subsequently, blood samples were collected and thyroid tissue was extracted for further analysis.

### 2.3. Histopathological Analysis

The thyroid tissue specimens underwent deparaffinization using xylene, dehydrated through an ethanol gradient, followed by staining with Hematoxylin and Eosin (HE) as well as Masson’s trichrome stain. After sealing with neutral gum, the specimens were air-dried. Subsequently, the specimens underwent examination using a light microscope, followed by photography and pathological analysis. The images of HE and Masson-stained sections were captured using a camera attached to a light microscope. To assess the severity of thyroid fibrosis, Image J software (Image J 2X, Bethesda, MD, USA) was employed for the quantification of Masson staining positive areas in the sections of thyroid tissue.

### 2.4. TUNEL Analysis

The apoptosis of thyroid cells was evaluated using the TUNEL assay. Prior to TUNEL staining, the thyroid tissue sections underwent deparaffinization, fixation, and permeabilization, then the DAPI counterstaining was also performed. The field of view was captured with a fluorescence microscope and the quantification of TUNEL-positive thyroid cells was recorded.

### 2.5. Immunohistochemical Analysis

The protein expression levels in thyroid tissue were detected by a immunohistochemical (IHC) staining kit. The paraffin sections were deparaffinized in water, followed by an antigen retrieval in a 98 °C water bath. Endogenous peroxidase was subsequently eliminated using a 3% hydrogen peroxide, and placed in an incubator and incubated for 30 min at 37 °C. After being cooled to room temperature, appropriate goat serum was added for blocking treatment for 30 min, and then appropriate dilutions of the primary antibody were added and incubated overnight at 4 °C (refer to Supplementary Table S4). Subsequently, the color was developed by adding the horseradish peroxidase reagent in a light-restricted environment. The cells were developed using DAB buffer and counterstained with hematoxylin before being fixed with neutral glue. Finally, observations were conducted using an optical microscope, and the average integrated optical density (IOD) value was quantitatively assessed by Image J software (Image J 2X, MD, USA).

### 2.6. Enzyme-Linked Immunosorbent Assay

The serum levels of thyroid hormones (TT_3_, FT_3_, TT_4_, FT_4_, TSH, and TG-Ab) and inflammation-related cytokines (IL-1β, IL-6, IL-10, and TNF-α) were quantitatively determined using an enzyme-linked immunosorbent assay (ELISA). The specific operational procedures strictly followed the instructions provided in the corresponding ELISA kit. Finally, the absorbance was measured at 450 nm using a microplate reader.

### 2.7. Statistic Analysis

SPSS 22.0 software (version 22.0; SPSS, Inc., Chicago, IL, USA) was used for statistical analysis of the data, and the experimental data were expressed as mean ± SD. The measurement data were initially assessed for normality, followed by the utilization of one-way ANOVA to compare groups. In cases where group variances were homogeneous, an LSD-*t* test was employed; whereas, Tamhane’s T2 test was employed in cases of non-homogeneous variances. The criterion for statistical significance was set at a significance level of *p* < 0.05.

## 3. Results

### 3.1. Serum 25(OH)D_3_ Levels and VDR Expression in Thyroid Tissue of Rats

**Figure 1:**
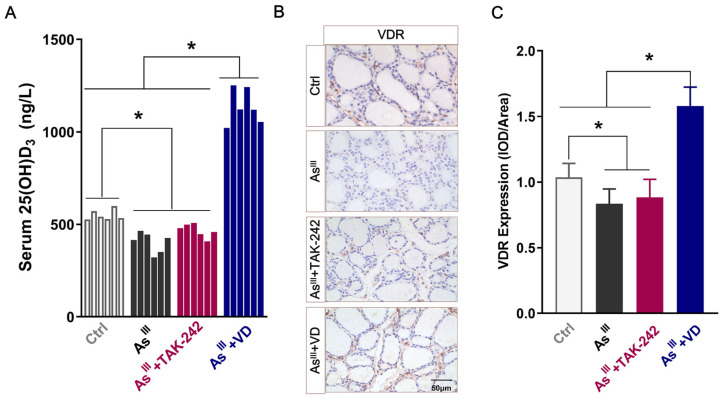
The alterations in serum 25(OH)D_3_ levels and VDR expression within the thyroid tissue of rats. (**A**) The serum 25(OH)D_3_ levels were quantified using the ELISA method. (**B**) The VDR expression in rat thyroid tissue in each group was detected by IHC (scale = 50 μm). (**C**) The average optical density of VDR was analyzed using Image J software (Image J 2X, MD, USA) (*n* = 6); * *p* < 0.05.

In order to investigate the effects of long-term arsenic exposure on VD levels in SD rats, ELISA was used to detect the serum secretion of 25(OH)D_3_ in each experimental group. Compared with the control group, chronic NaAsO_2_ exposure significantly decreased serum levels of 25(OH)D_3_ in rats (Figure 1A). TAK-242 intervention yielded comparable 25(OH)D_3_ levels to the arsenic-exposed group, whereas active VD supplementation significantly elevated serum 25(OH)D_3_ concentrations, surpassing those of the control, As^III^-exposed, and TAK-242-treated groups. The IHC analysis showed that the VDR protein level in As^III^-exposed group were significantly lower than those in the non-arsenic-exposed group. The levels did not show any significant alteration following the intervention of TAK-242; however, active VD intervention significantly enhanced the VDR protein expression levels in thyroid tissue of As^III^-exposed rats (Figure 1B,C). These findings suggest that chronic exposure to arsenic induced VD deficiency and down-regulated the VDR expression in rats.

### 3.2. Active VD-Inhibited Arsenite Exposure Triggered the Activation of the TLR4/NF-κB Signaling Pathway in Rats’ Thyroid Tissue

**Figure 2:**
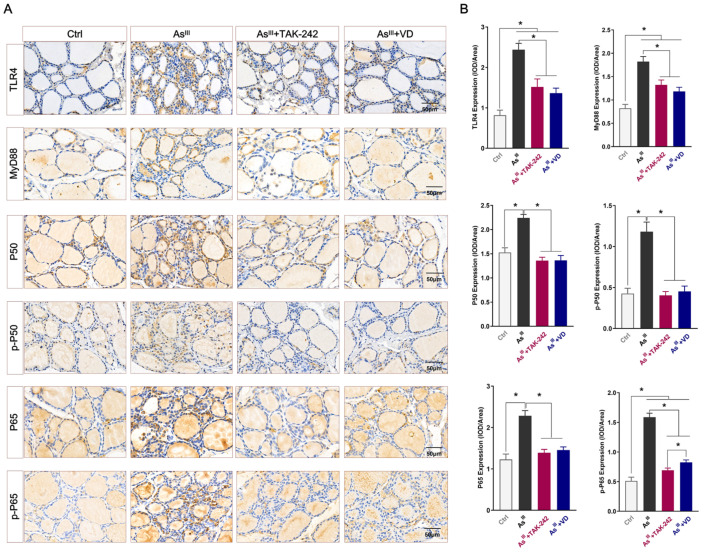
The protein expression of the TLR4/NF-κB signaling axis in rat thyroid tissue was assessed. (**A**) The expression levels of the TLR4/NF-κB signaling pathway-related proteins TLR4, MyD88, p50, p-P50, p65, and p-P65 in thyroid tissue of rats were detected by IHC (bar = 50 μm). (**B**) Image J software (Image J 2X, MD, USA) was selected to analyze the average optical density of TLR4, MyD88, p50, p-P50, p65, and p-P65 proteins (*n* = 6); * *p* < 0.05.

The expression of the TLR4/NF-κB signaling pathway-related proteins (TLR4, MyD88, p50, p-P50, p65, and p-P65) in rat thyroid tissue of each group were detected by IHC (Figure 2A), and Image J image software (Image J 2X, MD, USA) was further used for semi-quantitative analysis (Figure 2B). The results demonstrated that compared with the control group, TLR4 and MyD88 protein expression levels in thyroid tissue of the As^III^-exposed group were significantly increased. Additionally, the total protein levels of P50 and P65 were elevated in the thyroid tissue of the As^III^-exposed group, with significantly higher levels of phosphorylation (p-P50 and p-P65) expression. After TAK-242 intervention, there was a significant reduction in the total protein or phosphorylation levels of the aforementioned indicators compared to those observed in the As^III^-exposed group. Consistent with the intervention effect of TAK-242, the expression levels or phosphorylation of the aforementioned proteins in the thyroid tissue of rats subjected to active VD intervention exhibited a significant reduction compared to those observed in the As^III^-exposed group.

**Figure 3:**
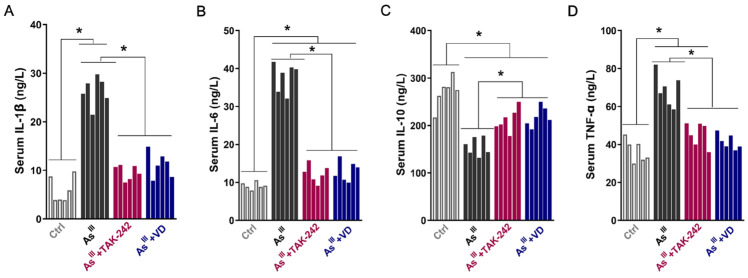
The serum levels of the TLR4/NF-κB signaling-related inflammatory cytokines, including (**A**) IL-1β, (**B**) IL-6, (**C**) IL-10, and (**D**) TNF-α in SD rats were detected (*n* = 6); * *p* < 0.05.

The serum levels of IL-1β, IL-6, IL-10, and TNF-α mediated by the TLR4/NF-κB inflammatory signal axis were further detected by ELISA. After As^III^ exposure for 36 weeks, compared to the control group, the levels of pro-inflammatory cytokines IL-1β, IL-6, and TNF-α in the serum of SD rats were significantly up-regulated (Figure 3). Conversely, there was a significant reduction in serum levels of anti-inflammatory factor IL-10. The administration of TAK-242 and active VD intervention effectively reduces the serum levels of pro-inflammatory cytokines IL-1β, IL-6, and TNF-α induced by chronic arsenic exposure in rats, while also moderately increasing the secretion level of IL-10, an anti-inflammatory cytokine.

### 3.3. Active VD Can Reduce the Apoptosis of Thyroid Cells Induced by Arsenic Exposure in Rats

**Figure 4:**
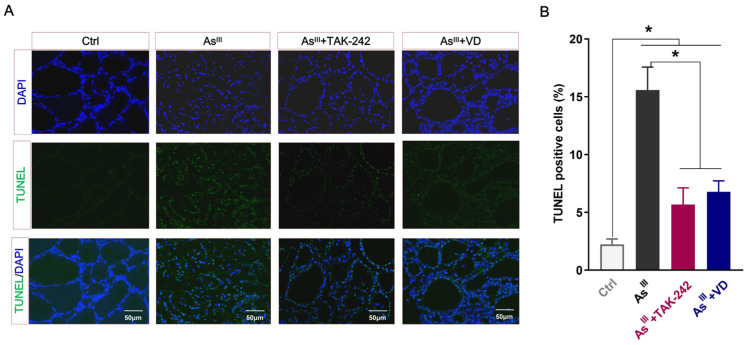
TUNEL staining and positive cells in thyroid tissues of rats. (**A**) Representative TUNEL staining plots were observed in sections of rat thyroid tissue from each experimental group. Thyroid cells labeled with TUNEL exhibit a green fluorescence, while DAPI-labeled nuclei displayed a blue fluorescence (bar = 50 μm). (**B**) TUNEL positive cell rate in rat thyroid tissue (%); * *p* < 0.05.

**Figure 5:**
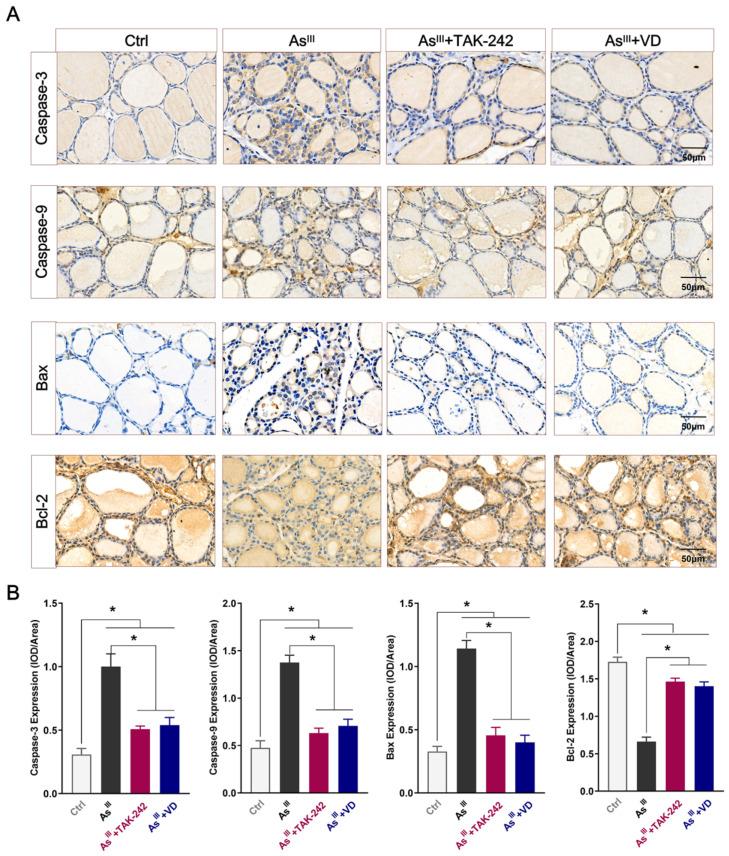
The apoptosis-related protein expression levels in the thyroid tissue of rats. (**A**) The protein levels of Caspase-3, Caspase-9, Bax, and Bcl-2 in different groups of rat thyroid tissue were evaluated by IHC (bar = 50 μm). (**B**) Mean optical density analysis of the above proteins was performed using Image J software (Image J 2X, MD, USA). (*n* = 6); * *p* < 0.05.

The intervention effect of active VD on apoptosis in rat thyroid cells exposed to NaAsO_2_ was assessed using TUNEL staining. The findings demonstrated a significant augmentation in the quantity of TUNEL-positive thyrocytes within the thyroid tissue of rats subjected to prolonged As^III^-exposure, which is consistent with our previously reported study findings [15]. However, compared to the As^III^-exposed group, both the TAK-242 and active VD intervention groups exhibited a significantly reduced number of TUNEL-positive thyrocytes (Figure 4A,B). Additionally, the IHC results depicted in Figure 5A,B demonstrate that the expression levels of apoptotic proteins Caspase-3, Caspase-9, and Bax were up-regulated, while the expression level of the anti-apoptotic protein Bcl-2 was down-regulated in thyroid tissue of the arsenic-exposed group compared with the control group. After TAK-242 and active VD intervention, the expression levels of Caspase-3, Caspase-9, and Bax in thyroid tissue of SD rats were significantly lower than those of the As^III^-exposed group. Conversely, there was a significant increase in the protein level of Bcl-2.

### 3.4. Active VD Reversed Arsenite Exposure Induced Abnormal Expression of THs Synthesis-Associated Proteins in the Thyroid Tissue of Rats

**Figure 6:**
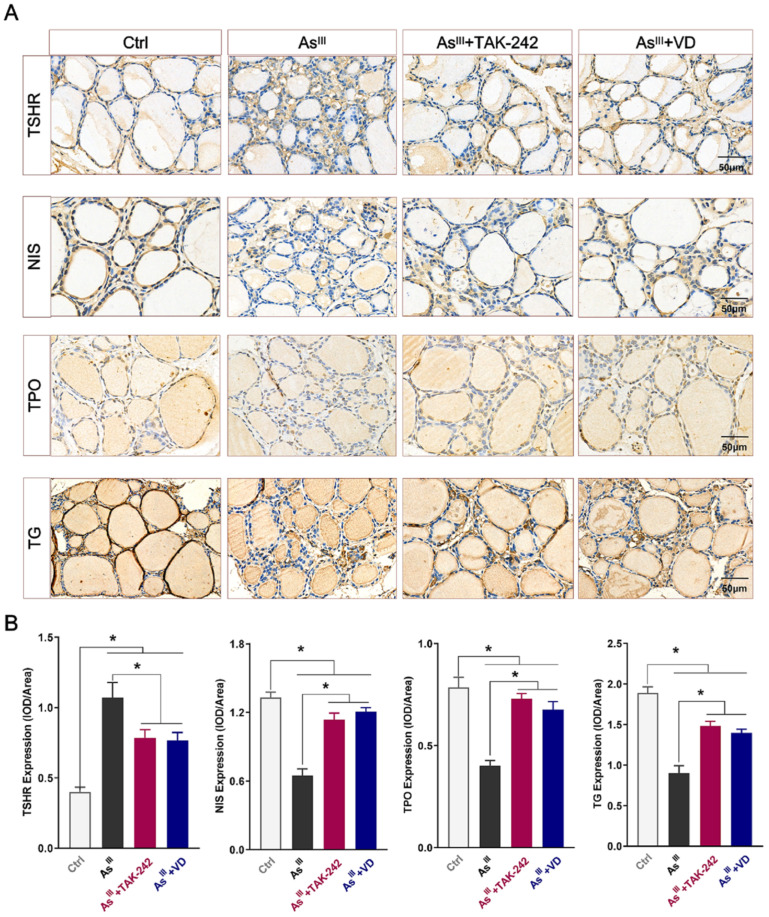
The expression levels of THs synthesis-related proteins in rat thyroid tissue. (**A**) The protein levels of TSHR, NIS, TPO, and TG in each group were detected by IHC (bar = 50 μm). (**B**) The optical density values of the above proteins were quantitatively analyzed by Image J software (Image J 2X, MD, USA) (*n* = 6). * *p* < 0.05.

The impact of chronic NaAsO_2_ exposure on the expression of proteins associated with THs synthesis (TSHR, NIS, TPO, and TG) was further investigated. IHC staining results revealed that the positive expressions of TSHR, NIS, TPO, and TG in thyroid tissue exhibited a light yellow or brown-yellow coloration. These expressions were predominantly localized within the cytoplasmic region of epithelial cells present in thyroid follicles (Figure 6A). Additionally, the average optical density of those proteins was further analyzed. Figure 6B demonstrates that compared with the control group, the expression levels of the TSHR protein in the thyroid tissue of the As^III^-exposed group were significantly up-regulated, while the protein expression levels of NIS, TPO, and TG were significantly down-regulated in the thyroid tissue of the As^III^-exposed group. However, after the intervention with TAK-242 and active VD, the protein expression levels of TSHR in the As^III^-exposed group was significantly decreased, while the protein expression levels of NIS, TPO, and TG were significantly increased compared with the As^III^-exposed group (Figure 6A,B). These findings of the aforementioned studies indicate that chronic As^III^ exposure can induce an aberrant expression of proteins related to THs synthesis, and the administration of active VD can effectively reverse these abnormal alterations.

### 3.5. Active VD Ameliorated Arsenite Exposure Induced Thyroid Injury and Dysfunction in Rats

**Figure 7:**
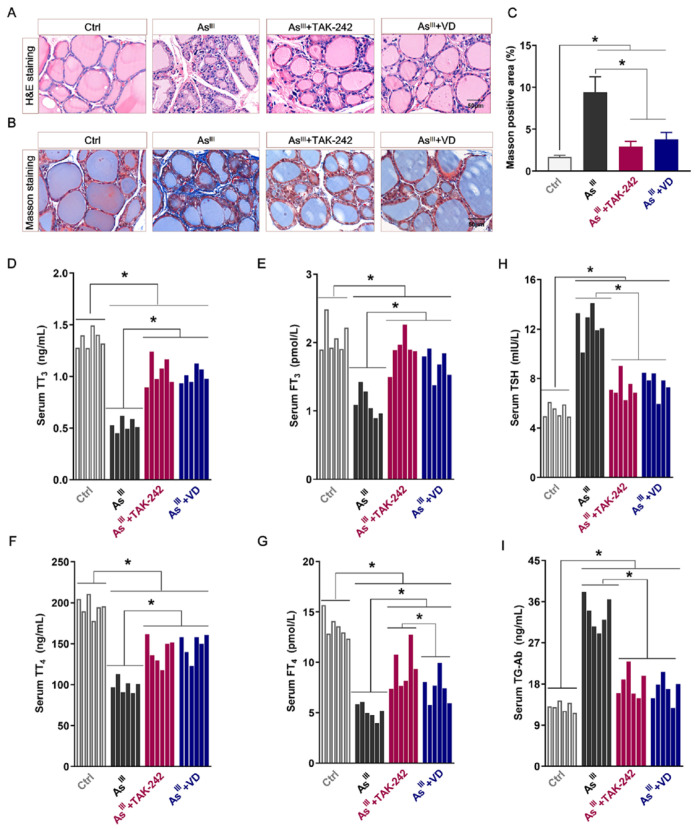
The histopathological staining of thyroid and serum THs secretion levels in rats. (**A**,**B**) The provided histopathological images depict rat thyroid tissue stained with HE as well as Masson’s trichrome (bar = 50 μm). (**C**) The percentage of Masson positive area in rat thyroid tissue. (**D**–**I**) The serum levels of TT_3_, FT_3_, TSH, TT_4_, FT_4_, and TG-Ab in each group (*n* = 6); * *p* < 0.05.

To comprehensively assess the intervention effect of active VD on chronic NaAsO_2_-induced damage to rats’ thyroid tissues, histological analysis was performed using HE staining and Masson staining. Simultaneously, ELISA was employed to evaluate serum levels of related THs (TT_3_, FT_3_, TSH, TT_4_, FT_4_, and TG-Ab). The histological analysis, as shown in Figure 7A–C, revealed that the thyroid follicles in the control rats exhibited a regular shape and consistent size. The colloid within the follicles appeared abundant and well filled with follicle cells, while no discernible positive fiber staining was observed. In contrast, prolonged exposure to As^III^ resulted in reduced thyroid nucleus size, severe damage to the follicular structure, inflammatory cell infiltration, collagen fiber hyperplasia, collagen positive staining area increase, and some fibrotic damage to the thyroid tissue. However, subsequent to the administration of TAK-242 and active VD, a noticeable reduction in the extent of inflammatory cell infiltration within thyroid tissue was observed. Furthermore, both the intensity and extent of collagen fiber coloration exhibited significant diminishment when compared to the As^III^-exposed group. Additionally, there was a substantial enhancement in the structural integrity of thyroid tissue.

The ELISA results showed that the levels of serum TT_3_, FT_3_, TT_4_, and FT_4_ in the long-term NaAsO_2_ treatment group were significantly decreased, while the levels of TSH and TG-Ab were on the contrary, showing a significant increase trend compared with the control group. Following TAK-242 and active VD intervention, the serum levels of TT_3_, FT_3_, TT_4_, and FT_4_ in rats were significantly increased compared with the arsenic exposure group, while the levels of TSH and TG-Ab were significantly decreased. The findings suggest that TAK-242 and active VD can antagonize thyroid tissue damage induced by long-term As^III^ exposure and improve thyroid function in rats.

## 4. Discussion

Being the largest endocrine gland in the human body, the thyroid gland plays a pivotal role in maintaining the optimal balance of THs. The results of our previous study demonstrate that exposure to As^III^ can induce structural damage and disrupt thyroid function in SD rats [15,16]. However, there is a lack of reports elucidating the precise molecular mechanism underlying thyroid damage induced by arsenic exposure, necessitating urgent further investigation.

In line with our previous findings, chronic arsenic exposure led to a significant decrease in the serum levels of TT_3_, FT_3_, TT_4_, and FT_4_, while concurrently elevating TSH levels. The secretion of TSH is under negative regulation by THs, in situations where serum T_4_ concentration decreases, there is a corresponding increase in the TSH concentration to ensure the stability of T_3_ levels [7]. Population epidemiological studies have also reported positive associations between arsenic exposure and serum TSH levels and negative associations with FT_4_ and FT_3_ [33]. Notably, our study also indicated a significant elevation in serum TG-Ab levels in rats following a prolonged exposure to arsenic. The studies have revealed that TSH not only regulates the levels of THs, but also undergoes regulation by TG. In response to arsenic stimulation, TG can stimulate lymphocytes to produce specific and highly concentrated Tg-Ab antibodies, thereby exerting a cytotoxic effect and inducing damage to thyroid cells [34]. Indeed, our TUNEL staining results also revealed that chronic exposure to arsenic could induce apoptosis in the thyroid cells of SD rats. As is widely acknowledged, the Caspase family and the Bc1-2 family are indispensable during apoptosis [35,36]. In line with our previous findings, chronic exposure to As^III^ resulted in the upregulation of pro-apoptotic proteins Caspase-3, Caspase-9, and Bax, and decreased the expression of the anti-apoptotic protein Bcl-2 in rats’ thyroid tissue [15].

The TSHR, NIS, TPO, and TG genes play a regulatory role in the formation of THs, and any dysfunction in these genes is closely linked to disorders in THs secretion. The present study found that the protein expression of TSHR was significantly increased, while the protein expression levels of NIS, TPO, and TG were markedly down-regulated in the thyroid tissue of rats after long-term exposure to arsenic. The upregulation of TSHR gene expression has also been reported to be potentially associated with hyperthyroidism [37]. Notably, arsenic exposure can activate inflammatory cytokines in the body, including TNF-α. Moreover, it has been reported that TNF-α activation leads to decreased NIS expression in thyroid cells [37]. The aforementioned studies and our results suggest that chronic As^III^ exposure may induce THs abnormal secretion by disrupting the expression of proteins involved in THs synthesis, with inflammatory factors potentially playing a crucial regulatory role in this process. Therefore, it is particularly necessary to understand the molecular mechanism of arsenic-induced thyroid injury from the perspective of immune-inflammatory responses.

TLR4 acts as a critical pattern recognition receptor, initiating downstream NF-κB activation, which leads to the secretion of inflammatory factors mediated by NF-κB and triggers a cascade of inflammatory responses [18]. Consequently, this leads to the secretion of inflammatory factors mediated by NF-κB and triggers a cascade of inflammatory responses [38,39]. We further investigated the expression levels of TLR4/NF-κB signaling axis-related proteins in rat thyroid tissue. The results revealed that chronic exposure to arsenic significantly induced phosphorylation of the TLR4 signaling pathway in rat thyroid tissue, resulting in elevated secretion levels of pro-inflammatory factors in serum. Population studies have consistently observed that the TLR4/NF-κB signaling pathway is frequently activated in patients with various thyroid diseases, and is closely related to thyroid dysfunction and the abnormal expression of THs synthesis-related genes [19,21,22,23]. The TLR4 inhibitor, TAK-242, effectively hinders the inflammatory signaling pathway mediated by TLR4 through specific binding to the cysteine 747 (Cys747) residue located in the TLR4 intracellular region [40]. The recent studies have shown that the intervention of TAK-242 can well block the TLR4-mediated immune inflammatory response, thereby impeding the progression of various diseases [41,42,43]. To further elucidate the mechanism of the TLR4/NF-κB signaling axis in arsenic-induced thyroid injury in SD rats, we administered the TAK-242 intervention to arsenic-exposed rats, resulting in significantly reduced expression levels and phosphorylation of the TLR4/NF-κB signaling axis-related proteins in thyroid tissue compared to the As^III^-exposed group. Simultaneously, the serum levels of pro-inflammatory factors IL-1β, IL-6, and TNF-α were significantly decreased. Compared with the As^III^-exposed group, the TAK-242 intervention group exhibited significant amelioration in the extent of thyroid tissue damage and abnormal THs secretion. The results confirm that the efficacy of the TAK-242 intervention can reduce arsenic-induced thyroid inflammatory injury and dysfunction by inhibiting the TLR4/NF-κB signaling pathway, thereby reducing the arsenic-induced inflammatory damage and dysfunction of the thyroid gland.

VD usually binds to the VDR through its active form of 1,25(OH)_2_D_3_, thereby directly or indirectly regulating the expression of genes associated with cell proliferation, differentiation, apoptosis, and other related processes [44]. The findings of this study unveiled that chronic arsenic exposure induces a down-regulation in the serum 25(OH)D_3_ levels and the VDR expression in thyroid tissue of SD rats. However, the active intervention of VD can significantly enhance the 25(OH)D_3_ levels of experimental rats exposed to arsenic and augment the VDR protein expression in thyroid tissue. More importantly, the supplementation of active VD effectively ameliorated the pathological injury to thyroid tissue, reduced inflammatory cell infiltration, and attenuated cell apoptosis induced by chronic arsenic exposure in SD rats. Consistent with our findings, VD_3_ intervention was observed to effectively mitigate inflammatory cell infiltration in rat thyroid tissue and ameliorate the extent of thyroid injury in rats with osteoporosis [45]. Another study conducted on an animal model of experimental autoimmune thyroiditis (EAT) demonstrated that intervention with 1,25(OH)_2_D_3_ effectively ameliorated thyroid pathological changes and reversed the cytokine imbalance and autoantibody production in EAT rats [46]. We further revealed a significant elevation in serum levels of TT_3_, FT_3_, TT_4_, and FT_4_, accompanied by a notable reduction in TSH and TG-Ab levels in rats exposed to long-term arsenic exposure after active VD supplementation compared to rats solely exposed to arsenic. Additionally, in comparison to the group exposed to arsenic, there was a significant down-regulation of the TSHR protein expression in rats’ thyroid tissue, while the protein expression levels of NIS, TPO, and TG were up-regulated. Results of a systematic review and meta-analysis indicate that VD supplementation effectively reduces TG-Ab and TSH levels in the serum of patients with HT, while simultaneously increasing the levels of FT_3_ and FT_4_ [47]. The in vivo animal study demonstrates that mice with VD deficiency may develop persistent hyperthyroidism following immunization with thyroid-stimulating hormone receptor (TSHR), whereas mice with adequate levels of VD do not exhibit such symptoms [48]. The above findings suggest that active VD intervention can effectively regulate thyroid dysfunction caused by arsenic exposure in SD rats. However, the molecular mechanism by which VD improves arsenic-induced thyroid damage is still unclear.

VD plays a crucial role in modulating the immune system by suppressing the synthesis of relevant proteins within the TLR4/NF-KB signaling pathway and pro-inflammatory cytokines such as IL-6, IL-1β, and TNF-α, while promoting the generation of the anti-inflammatory cytokine IL-10 [29,49]. The present study further examined the protein expression levels associated with the TLR4/NF-κB signaling axis and assessed the inflammatory cytokines secretion levels mediated by active VD intervention in rats’ thyroid tissue. The findings demonstrated that active VD intervention well mitigated the activation of the TLR4/NF-κB signaling pathway in thyroid tissue caused by long-term arsenic exposure, subsequently reducing the pro-inflammatory factors secretion levels in serum. Building upon our studies utilizing TAK-242 intervention, which have established the inhibitory role of the TLR4/NF-κB signaling pathway in arsenic-induced thyroid damage in rats, the present findings suggest that active VD may mitigate structural and functional disorders of the rats’ thyroid gland resulting from long-term arsenic exposure by regulating the TLR4/NF-κB inflammation-related signaling pathway.

## 5. Conclusions

In general, based on the previous establishment of an animal model of thyroid injury and dysfunction caused by long-term arsenic exposure in SD rats, the present study further employed the intervention effect of active VD on arsenic-induced thyroid injury. The results confirmed that active VD intervention significantly suppressed the activation of the TLR4/NF-κB signaling axis in the thyroid tissue induced by prolonged arsenic exposure in SD rats. Additionally, this intervention reduced pro-inflammatory factor secretion levels, effectively mitigated structural damage to the thyroid tissue resulting from arsenic exposure, and regulated proteins expression involved in THs synthesis. Ultimately, it improved serum THs secretion levels and restored thyroid function. The outcomes of this research offer novel insights on the potential utilization of active VD in targeted prevention and treatment of endocrine disorders, particularly thyroid dysfunction resulting from arsenic exposure. However, due to the limitations of in vivo animal observations, further investigations are imperative for experimental verification of the VD intervention effect on populations exposed to arsenic, particularly those exhibiting endocrine abnormalities.

## Data Availability

The data presented in this study are available on request from the corresponding author.

## Supplementary Materials

The following supporting information can be downloaded at https://www.mdpi.com/article/10.3390/toxics12120887/s1; Table S1. Details regarding the reagents and instruments utilized in this study; Table S2. The primary raw material of feed ingredients utilized in this study; Table S3. The dietary composition of the feed ingredients utilized in this study; Table S4. List of primary antibodies and ELISA kits utilized in this study.
