# Supplementary material for: Active Vitamin D Ameliorates Arsenite-Induced Thyroid Dysfunction in Sprague–Dawley Rats by Inhibiting the Toll-like Receptor 4/NF-KappaB-Mediated Inflammatory Response
Source: Toxics. 2024 Dec 6;12(12):887. doi: 10.3390/toxics12120887 (PMC11728788; doi:10.3390/toxics12120887)
Supplement: Supplementary file 1 [file toxics-12-00887-s001.zip › toxics-3340051-supplementary.pdf]

Table Sl. Details regarding the reagents and instruments utilized in this study

Reagents or instruments	Producer and nation
Sprague-Dawley (SD) rats	Liaoning, China (SCXK-LN2015-0001)
Calcitriol (purity 99. 46%)	MCE , China
Sodium Arsenite ($NaAsO_2$)	Sigma, MO, USA
TAK-242	MCE, SNY, USA
H&E staining kit	Solarbio, Beijing, China
Masson staining kit	Solarbio, Beijing, China
Immunohistochemical (IHC) staining kit	Solarbio, Beijing, China
Terminal deoxynucleotidyl transferase-mediated dUTP nick-end labeling (TUNEL) assay	Sigma-Aldrich, USA
Microplate reader	ThermoFisher, MA, USA
Light microscope	Nikon, Tokyo, Japan
Fluorescence microscope	Zeiss, LSM700B, Germany
Image J software	Image J 2X, MD, USA
GraphPad Prism 8.0 software	Graphpad Software Inc., CA, USA
SPSS 22.0 software	SPSS Inc., IL, USA

Table S2. The primary raw material of feed ingredients utilized in this study

Nutrients	Sources of nutrients
Protein	Soybean meal, fish meal, brewer's yeast powder
Fat	vegetable oil
Carbohydrates	Corn, flour
Fiber	Bran
Minerals	Calcium hydrogen phosphate, stone powder, food salt, iron, copper, manganese, zinc, etc
Vitamin	VA, VD, VE, VK, B vitamins, choline, etc
Nutrients	Sources of nutrients
Protein	Soybean meal, fish meal, brewer's yeast powder
Fat	vegetable oil
Carbohydrates	Corn, flour

Table S3. The dietary composition of the feed ingredients utilized in this study

Indicators	Guaranteed value of Main Nutrients (g/kg)
Moisture	⩽100
Crude protein	⩾200
Crude fat	⩾40
Crude fibre	⩽50
Crude ash	⩽80
Calcium	⩾10-18
Phosphorus	⩾6-12
Lysine	⩾8.2
Methionine+ cystine	⩾7.8
Arginine	⩾9.9

Table S4. List of primary antibodies and ELISA kits utilized in this study

**Primary Antibody/ELISA kits**	**Producer and nation**
TLR4 antibody	Santa Cruz, California, USA
MyD88 antibody	Proteintech, Wuhan, China
p-P50 antibody	Absin,Shanghai, China
p-P65 antibody	SAB, Greenbelt, USA
p50 antibody	Proteintech, Wuhan, China
p65 antibody	SAB, Shanghai, China
Caspase-3 antibody	Proteintech, Wuhan, China
Caspase-9 antibody	Proteintech, Wuhan, China
Bax antibody	Affinity, Jiangsu, China
Bcl-2 antibody	Proteintech, Wuhan, China
TSHR antibody	Proteintech, Wuhan, China
NIS antibody	Proteintech, Wuhan, China
TPO antibody	Immunoway, Beijing, China
TG antibody	Abcam, Cambridge, UK
IL-1β ELISA kit	Mlbio, Shanghai, China
IL-6 ELISA kit	Mlbio, Shanghai, China
IL-10 ELISA kit	Mlbio, Shanghai, China
TNF-α ELISA kit	Mlbio, Shanghai, China
Total Triiodothyronine(TT_3) ELISA kit	Mlbio, Shanghai, China
Total Thyroxine(TT_4) ELISA kit	Mlbio, Shanghai, China
Free Triiodothyronine(FT_3) ELISA kit mlbio	Mlbio, Shanghai, China
Free Thyroxine(FT_4) ELISA kit	Mlbio, Shanghai, China
Thyroid stimulating hormone (TSH) ELISA kit	Mlbio, Shanghai, China
Anti-thyroglobulin Antibodies(TG-Ab） ELISA kit	Mlbio, Shanghai, China
